# Biocompatibility of Pit and Fissure Sealants: Scoping Review of In Vitro and In Vivo Evidence

**DOI:** 10.3390/dj14070425

**Published:** 2026-07-10

**Authors:** Marija Badrov, Karmela Džaja, Barbara Badrov, Ana Glavina, Antonija Tadin

**Affiliations:** 1 Department of Restorative Dental Medicine and Endodontics, Study of Dental Medicine, School of Medicine, University of Split, 21000 Split, Croatia; karmela.dzaja@mefst.hr; 2 Division of Dental Medicine, University Hospital Center of Split, 21000 Split, Croatia; glavina2014@gmail.com; 3 Split-Dalmatia County Pharmacy, 21204 Dugopolje, Croatia; barbara44badrov@gmail.com; 4 Department of Oral Medicine, Study of Dental Medicine, School of Medicine, University of Split, 21000 Split, Croatia

**Keywords:** pit and fissure sealants, dental materials, biocompatibility, cytotoxicity, genotoxicity, caries prevention

## Abstract

**Objectives:** This scoping review summarized the evidence on the biocompatibility of pit and fissure sealants, focusing on cytotoxicity, genotoxicity, and overall biological safety of commercial and experimental materials evaluated in vitro and in vivo. **Methods:** Following the PRISMA-ScR guidelines, eligibility was defined using the Population, Concept, and Context (PCC) framework: the population comprised cell cultures, animal models, or human participants exposed to sealants; the concept was biocompatibility, including cytotoxicity, genotoxicity, and inflammatory or tissue response; and the context encompassed commercial and experimental pit and fissure sealants used in preventive dentistry, particularly in pediatric populations. PubMed and Scopus platforms were searched without restrictions on publication year or language. Studies assessing biocompatibility (cytotoxicity, genotoxicity, inflammatory or tissue response) in cell cultures, animal models, or humans were eligible; those evaluating only clinical efficacy were excluded. Two reviewers independently performed study selection and data extraction. **Results:** Of 406 records (291 after deduplication), 10 studies were included—nine in vitro and one in vivo. Resin-based sealants predominated, mainly assessing residual monomers (TEGDMA, Bis-GMA) and their effects on fibroblasts, keratinocytes, periodontal ligament cells, and buccal epithelial cells. TEGDMA was released most frequently, whereas Bis-GMA showed the highest cytotoxicity. Experimental sealants containing nano-calcium fluoride, calcium phosphate, bioactive glass, or antibacterial monomers generally showed favorable biocompatibility, although high additive concentrations reduced cell viability. The single in vivo study reported good biocompatibility without significant genotoxicity. **Conclusions:** Pit and fissure sealants generally show acceptable biocompatibility and remain safe for caries prevention, although the biological response depends on composition, degree of polymerization, and residual monomer release. Further standardized long-term in vivo research is needed, particularly in pediatric populations.

## 1. Introduction

Dental caries is a biofilm-mediated chronic disease resulting from complex interactions among microbial communities including *Streptococcus mutans*, *Actinomyces* spp., and *Lactobacillus* spp., dietary habits, host susceptibility, and time, leading to disruption of mineral homeostasis and the formation of lesions in both primary and permanent teeth [1]. Its prevalence is increasing worldwide, particularly among children and adolescents [2]. Caries lesions most often develop on the occlusal surfaces of premolars and molars during eruption, when immature enamel, limited post-eruptive mineralization, and deep pits and fissures favor bacterial colonization and acid production [2]. In clinical practice, multiple preventive strategies are available, including fluoride-based agents such as gels, toothpastes, mouthrinses, and varnishes, as well as pit and fissure sealants to protect vulnerable occlusal surfaces [3].

Pit and fissure sealants were first introduced into clinical practice in the 1960s as a preventive strategy for protecting vulnerable occlusal surfaces [4]. Application of pit and fissure sealants forms a protective interface over susceptible enamel, restricting substrate availability to cariogenic microorganisms and thereby suppressing biofilm maturation and metabolic activity [5]. The most used materials for pit and fissure sealing are resin-based formulations and glass ionomer cement. Resin-based sealants typically consist of urethane dimethacrylate (UDMA) or bisphenol A-glycidyl methacrylate (Bis-GMA) monomers, whereas glass ionomer sealants are composed of fluoroaluminosilicate glass powder combined with an aqueous polyacrylic acid solution [4]. Newer materials on the market aim to combine the advantages of resin-based and glass ionomer sealants. For instance, compomers are resin-based materials that release fluoride, while resin-modified glass ionomers incorporate resin components into glass ionomer formulations to enhance their mechanical properties [6].

A 2017 systematic review concluded that resin-based sealants applied to permanent molars effectively prevent caries in children and adolescents, with moderate-quality evidence showing a 51% reduction compared with unsealed surfaces at follow-up of up to 48 months. Evidence for glass ionomer sealants and for comparisons among different sealant types was insufficient [6]. However, clinical practice guidelines published in 2017 recommend the use of pit and fissure sealants to prevent and arrest non-cavitated carious lesions. These guidelines indicate that both resin-based and glass ionomer sealants are effective in preventing caries and slowing lesion progression on the occlusal surfaces of primary and permanent molars [7]. A comprehensive review published in 2018 highlighted the clinical benefits of pit and fissure sealants [8]. The main recommendations indicate that sealing the pits and fissures of primary and permanent teeth is both safe and effective for preventing caries and arresting early lesions. Long-term success, however, depends on regular dental checkups and the timely renewal of sealants when necessary [8].

Despite their widespread use in dentistry, particularly in pediatric populations, relatively few studies have investigated the biocompatibility and safety of these materials in relation to oral tissues. Because dental materials remain in prolonged direct contact with oral tissues and cannot be removed by patients, continuous evaluation of their biological effects is essential, even for materials used clinically for decades [9]. Concerns about resin matrix components have grown with the expanding use of polymer-based materials in the oral cavity, as composite resins may release residual components over time despite their clinical acceptance [10]. This release occurs via two main mechanisms: elution of unreacted monomers and additives after polymerization, and hydrolytic or enzymatic degradation of the polymer matrix, forming oligomers and monomers [11]. Inadequate polymerization may leave approximately 25–50% of methacrylate monomers unreacted, allowing their diffusion through dentinal tubules toward the pulp [12]. Triethylene glycol dimethacrylate (TEGDMA) is consistently identified as the predominant monomer released from pit and fissure sealants [13,14,15], while some studies also report notable levels of Bis-GMA [16,17]. Residual monomers may exert cytotoxic effects via oxidative stress, including depletion of intracellular glutathione and increased reactive oxygen species (ROS) production, potentially leading to DNA damage [12]. Other biologically active substances, such as formaldehyde, initiator fragments, and filler-derived ions, may also be released. Cytotoxicity is generally highest before and immediately after polymerization and lower, though still detectable, in fully cured materials, while long-term biodegradation contributes further degradation products, underscoring the need to assess both short- and long-term biocompatibility [11]. Fluoride-containing pit and fissure sealants promote remineralization and exert antibacterial effects; however, fluoride has also been reported to have a potential genotoxic effect [18].

A systematic review published in 2008 examined the potential effects of bisphenol A (BPA) in dental sealants. The review concluded that pit-and-fissure sealants do not pose a significant risk of BPA exposure. To further minimize any potential BPA-related effects, clinicians are advised to apply a mild abrasive such as pumice, have patients rinse with water, or thoroughly wash the sealant surface after placement [19]. Furthermore, the 2016 Evidence-based Clinical Practice Guideline for the use of pit-and-fissure sealants reported ongoing concerns regarding potential adverse effects of dental sealants, primarily related to BPA. Although BPA may exhibit estrogen-like activity, current evidence suggests that the transient release of small amounts does not pose a significant biological risk [20].

Recent evidence has raised concerns regarding the biological safety of bisphenol A (BPA) and its analogues, even at low exposure levels. A scoping review demonstrated that BPA exposure is associated with adverse effects on oocyte quality, including disrupted meiotic processes, spindle abnormalities, and impaired developmental competence, across in vitro, in vivo, and human studies [21]. Notably, these effects were observed even at doses considered biologically safe, while BPA substitutes showed comparable detrimental effects, highlighting potential limitations in the safety of current resin-based materials [21]. Bisphenol A (BPA), derived from BPA-based monomers such as Bis-GMA, has been identified as a potential endocrine disruptor with estrogen-like activity [12]. Additionally, resin-based dental materials are not considered a major contributor to systemic BPA exposure, although the issue remains under investigation [11].

The literature on primary in vivo and in vitro investigations evaluating the cytotoxic effects of pit and fissure sealants on oral tissues and cells remains limited and heterogeneous. Despite their widespread use in dental practice, particularly in the pediatric population, there is still no comprehensive synthesis of their biological effects. To date, no systematic review or meta-analysis has specifically addressed the in vitro and in vivo biocompatibility, cytotoxicity, or genotoxicity of these materials. This lack of consolidated evidence is particularly important given the frequent exposure of children to pit and fissure sealants during routine preventive dental care, where safety considerations are of critical relevance. In addition, existing studies vary considerably in design, outcomes, and methodological quality, which further limits direct comparison of results. Therefore, the present scoping review aims to map and critically summarize the available literature on the cytotoxic, genotoxic, and overall biocompatibility of pit and fissure sealants, identifying key gaps in knowledge and providing a structured overview of current evidence relevant to clinical practice in pediatric dentistry.

## 2. Materials and Methods

### 2.1. Eligibility Criteria

This scoping review focused on both in vitro and in vivo studies, including in vitro investigations using cell lines and tissue cultures, as well as in vivo research involving animal models and human studies. All available studies assessing the biocompatibility of dental fissure sealants were considered for inclusion, including but not limited to investigations of cytotoxicity, genotoxicity, inflammatory response, and tissue response. The review encompassed fissure and pit sealant materials, including resin-based, glass ionomer, and resin-modified glass ionomer sealants, as well as other emerging materials. Studies were eligible for inclusion regardless of clinical setting or geographic location, with no restrictions on study duration, language, or publication type. The following exclusion criteria were applied: studies focusing solely on clinical efficacy without assessing biocompatibility; conference abstracts without full text; studies on adhesive materials not specifically used as fissure sealants; and opinion pieces, letters, or editorials lacking original data.

Eligibility was structured according to the Population–Concept–Context (PCC) framework: the population comprised cells, tissues, or patients exposed to pit and fissure sealants; the concept was biocompatibility, including cytotoxicity, genotoxicity, inflammatory response, and tissue response; and the context was the in vitro and in vivo evaluation of commercial and experimental sealant materials, with particular relevance to pediatric preventive dentistry. Accordingly, the review addressed the following questions: (1) What is the available evidence on the biocompatibility of pit and fissure sealants? (2) Which material components and exposure conditions are associated with cytotoxic or genotoxic effects?

### 2.2. Information Sources and Search Strategy

A comprehensive search was conducted to identify all relevant studies, regardless of language or publication date. Studies for which full texts were not directly accessible during the initial database search were obtained subsequently through supplementary search strategies, including manual searching and alternative access routes. Additional information sources included the grey literature from relevant institutional repositories and, where applicable, technical documentation provided by manufacturers. The databases consulted were PubMed and Scopus, selected for their comprehensiveness and reliability. The methodology of this scoping review adhered to the PRISMA-ScR guidelines [22,23]. Although a protocol was developed a priori to guide the review process, the review was not prospectively registered because PROSPERO does not accept scoping reviews.

A detailed search strategy was developed for MEDLINE and Scopus using a combination of controlled vocabulary and free-text terms (Table 1). The database searches were last run on 28 March 2026.

### 2.3. Data Selection Process and Data Items

**Figure 1:**
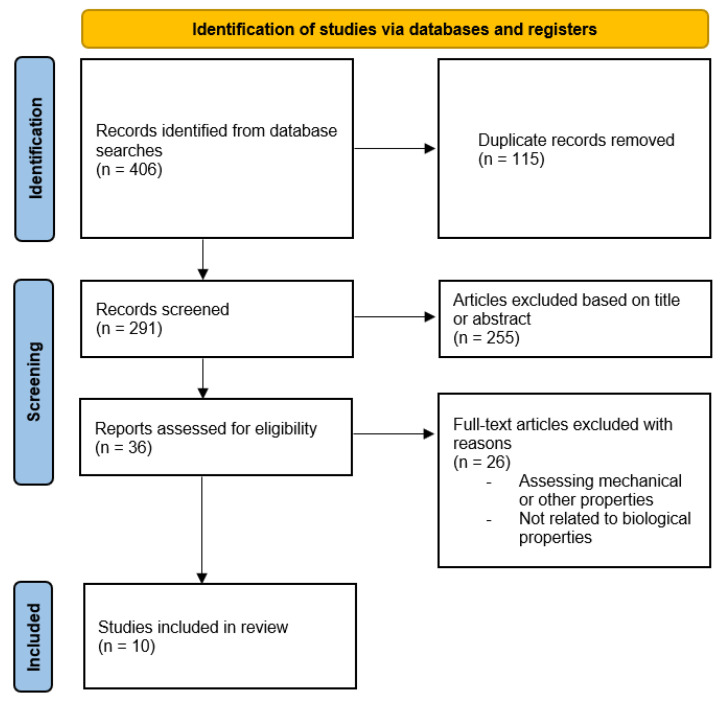
PRISMA-ScR flow diagram of the study identification and selection process.

In the initial phase of the study selection process, a total of 406 records were identified through database searching. Following the removal of duplicate records (n = 115), 291 unique records were retained for further screening. Title and abstract screening was subsequently performed independently by three reviewers (M.B., K.DŽ., and B.B.) in accordance with the predefined eligibility criteria. Any discrepancies or disagreements between reviewers were resolved through discussion and, when necessary, consultation with two additional reviewers (A.T. and A.G.) to reach a consensus. Following the initial screening phase, 36 articles were selected for full-text assessment. Full-text screening was also conducted independently by the same three reviewers to ensure consistency and minimize selection bias. Studies that did not meet the inclusion criteria were excluded, with reasons for exclusion systematically documented and are presented in the PRISMA flow diagram (Figure 1). At the full-text eligibility stage, studies were included if they specifically investigated the biocompatibility of pit and fissure sealants, as assessed through relevant biological endpoints, including cytotoxicity, genotoxicity, or *other* in vitro or in vivo cellular responses. Studies were excluded if they did not address biological outcomes related to biocompatibility. In particular, articles focusing solely on mechanical properties, polymerisation kinetics, material composition, adhesion, or other physicochemical characteristics of pit and fissure sealants, without accompanying evaluation of biological or cellular effects, were deemed ineligible for inclusion. The overall study selection process is further illustrated in the PRISMA flow diagram, which provides a transparent overview of identification, screening, eligibility, and inclusion phases.

### 2.4. Data Extraction

Data were extracted into a standardized Excel sheet form. Extracted information included study characteristics such as author, year and study design. Material characteristics captured the type of sealant, commercial name, and manufacturer, chemical composition, and application method. Methodological details were recorded for in vitro studies, including cell type, assay methods, exposure time, and concentrations, and for in vivo studies, including animal model, study duration, and administration route. Outcomes included the measured biocompatibility parameters, main findings, statistical significance, and any reported adverse effects.

### 2.5. Synthesis of Results:

Extracted data were summarized descriptively and organized according to study design, sealant type, biological outcome, and principal findings. No meta-analysis was undertaken because of methodological heterogeneity.

## 3. Results

The search identified 406 articles, and after removing duplicates, 291 articles were assessed. Based on the title and abstract, 255 articles were excluded. After applying the eligibility criteria, 36 full texts were analyzed, and upon review, 10 articles were included in the study (Figure 1). Included studies are shown in Table 2.

To make the principal outcomes easier to compare at a glance, Table 3 summarizes the key biocompatibility findings of each included study.

Among the included studies, nine were in vitro investigations [13,14,24,25,26,28,29,30,31], while only one study was conducted in vivo [27]. The predominance of laboratory-based studies highlights the limited availability of clinical evidence regarding the biocompatibility of pit and fissure sealants. Five studies evaluated the biological properties of commercially available fissure and pit sealants [13,14,27,30,31], whereas the remaining studies focused on the development of novel experimental sealants and compared their cytotoxicity and biological performance with existing commercial materials [24,25,26,28,29]. Most experimental materials were resin-based formulations modified with bioactive or antibacterial additives, including nano-calcium fluoride (nCaF_2_), bioactive glass (BAG), polylysine (PLS), monocalcium phosphate monohydrate (MCPM), antibacterial monomers such as DMAHDM, and chitosan/fluoride microparticles.

The most frequently used biological assays were MTT assays [24,25,29], followed by XTT [14], WST-1 [28], SRB [26], LDH assay [31], agar overlay assay [30], trypan blue exclusion [13], and micronucleus assay for genotoxicity evaluation [27]. Cell models included fibroblasts, keratinocytes, periodontal ligament cells, gingival fibroblasts, dental pulp cells, and buccal epithelial cells, reflecting the diversity of tissues potentially exposed to sealant-derived components.

Most studies demonstrated that cytotoxicity was strongly influenced by sealant composition, particularly the presence and concentration of residual monomers and additives. TEGDMA was consistently identified as one of the most commonly released monomers, while Bis-GMA exhibited the highest cytotoxic potential. Fei et al. [25] reported that toxicity followed the order Bis-GMA > DMAHDM > TEGDMA, while nano-CaF_2_ did not show toxic effects at the tested concentrations. Similarly, Furche et al. [14] detected significant elution of TEGDMA and identified Fissurit^®^ F as the most cytotoxic material among the tested commercial sealants.

Several studies showed that increased concentrations of certain additives could negatively affect cell viability. Garcia et al. [26] found that incorporation of 5 wt.% TAT into BisGMA/TEGDMA resin significantly reduced keratinocyte viability, whereas 2.5 wt.% showed no significant difference compared with controls. Lai et al. [28] observed cell necrosis in 3T3 fibroblasts exposed to 4% chitosan/fluoride microparticles, likely due to excessive fluoride concentration. In contrast, Lertwisitphon et al. [24] demonstrated that formulations containing calcium phosphate, bioactive glass, and polylysine showed favorable biocompatibility, with F1 exhibiting the highest cell viability.

Comparative studies of commercial sealants also showed substantial differences in cytotoxic potential. Koulaouzidou et al. [13] reported that BeautiSealant, Clinpro, and Grandio Seal caused the greatest reduction in fibroblast viability, while Helioseal Clear and Conseal F showed comparatively lower cytotoxicity. Zingler et al. [30] found that unfilled resin-based sealants exhibited the highest cytotoxicity, followed by filled resin-based sealants, resin-modified glass ionomer sealants, and silicone-based sealants, which demonstrated the lowest cytotoxic potential.

Polymerization conditions also influenced biological outcomes. Wegehaupt et al. [31] showed that shortening light-curing time while maintaining equivalent energy density did not increase cytotoxicity, suggesting that optimized polymerization protocols may reduce clinical concerns regarding incomplete curing.

The only included in vivo clinical study by Gavić et al. [27] evaluated cytotoxic and genotoxic effects of Helioseal F^®^, Equia Fil^®^, and Constic^®^ using buccal epithelial cells over a 90-day period. Overall, all tested materials demonstrated good biocompatibility. Only Helioseal F^®^ showed a statistically significant increase in condensed chromatin after 90 days, while no significant increase in micronuclei or other genotoxic markers was observed for the other materials.

Overall, the available evidence suggests that most fissure and pit sealants demonstrate acceptable short-term biocompatibility, although resin-based materials containing higher concentrations of unreacted monomers, particularly Bis-GMA and TEGDMA, may exhibit greater cytotoxic potential.

## 4. Discussion

Biocompatibility remains one of the fundamental requirements for all dental materials, particularly for pit and fissure sealants that are placed in direct contact with enamel surfaces and remain exposed to the oral environment for prolonged periods. Unlike removable preventive agents, sealants remain intraorally for years, and patients, especially children, are continuously exposed to their components and degradation products. Although these materials are widely accepted in preventive dentistry and are considered clinically safe, concerns regarding the release of residual monomers, fluoride-related biological effects, and possible genotoxicity continue to justify ongoing investigation. This is particularly important in pediatric dentistry, where sealants are routinely applied during the early eruption period of permanent molars, often representing one of the first restorative or preventive interventions in a child’s life.

The present scoping review aimed to summarize the available evidence regarding the cytotoxicity, genotoxicity, and overall biocompatibility of pit and fissure sealants. The results demonstrated that most of the available evidence originates from in vitro studies, while clinical in vivo studies remain extremely limited. Among the ten included studies, only one clinical study directly evaluated biological effects in human subjects, while the remaining studies were laboratory-based investigations using fibroblasts, keratinocytes, periodontal ligament cells, pulp cells, and other cell models. This imbalance between laboratory and clinical evidence highlights a significant gap in the literature and limits the ability to directly translate findings into long-term clinical recommendations.

Resin-based sealants were the most frequently investigated materials, which reflects their dominant use in clinical practice due to superior retention rates and strong evidence supporting their caries-preventive effectiveness. Their excellent micromechanical retention and long-term clinical performance make them the preferred material for sealing permanent molars, particularly in children at high caries risk [6,7]. However, the same resin matrix responsible for these favorable mechanical properties also represents the main source of biological concern. Resin-based sealants typically contain methacrylate monomers such as Bis-GMA, TEGDMA, UDMA, and HEMA, which may remain partially unreacted after polymerization and subsequently leach into the oral environment [11].

Incomplete polymerization remains one of the most important factors influencing cytotoxicity. It has been reported that approximately 25–50% of methacrylate monomers may remain unreacted after light curing, depending on material composition, curing protocol, oxygen inhibition, and clinical accessibility [12]. These residual monomers may diffuse through enamel microspaces, dentinal tubules, and saliva, reaching gingival tissues, pulpal tissues, and even systemic circulation in small amounts. The biological consequences include oxidative stress, depletion of intracellular glutathione, increased production of reactive oxygen species, mitochondrial dysfunction, inflammatory signaling, and DNA damage [13,14]. These mechanisms explain why cytotoxicity is often highest immediately after polymerization and decreases over time, although degradation products may continue to be released during long-term clinical service.

A pattern that recurs across the included studies is the dissociation between how much of a monomer is released and how toxic it is. Owing to its low molecular weight and hydrophilicity, TEGDMA is eluted in the largest amounts and over the longest periods, yet Bis-GMA repeatedly emerges as the more intrinsically cytotoxic species, exerting stronger effects even at lower concentrations [14,25]. Biologically, this distinction matters more than the absolute quantity eluted: lowering TEGDMA reduces total leachate, but residual Bis-GMA can still drive cellular injury. This helps explain why materials with broadly similar monomer release can nonetheless differ appreciably in measured cytotoxicity, and it argues for evaluating individual monomer identity rather than total elution alone.

This finding is particularly relevant because Bis-GMA is synthesized from bisphenol A derivatives, raising concerns regarding potential endocrine-disrupting effects. BPA has been widely discussed in dentistry due to its estrogen-like activity and potential reproductive toxicity. Earlier systematic reviews concluded that pit and fissure sealants do not pose a significant risk of BPA exposure and that the transient release of trace amounts is unlikely to produce clinically relevant adverse effects [19]. Similarly, the ADA clinical guideline stated that current evidence does not support significant biological risk from BPA released by dental sealants [20]. However, more recent evidence regarding BPA and its analogues suggests that even low-dose exposure may influence reproductive health, oocyte quality, meiotic stability, and developmental competence [21]. Although dental materials are not considered the main source of systemic BPA exposure, the cumulative effect of repeated exposure from multiple resin-based restorations during childhood remains insufficiently understood and deserves further investigation.

An important finding of this review is that attempts to improve antibacterial and remineralizing properties of sealants may significantly influence their biological behavior. Newer generations of sealants increasingly incorporate fluoride-releasing agents, antibacterial monomers, calcium phosphate systems, bioactive glass, and peptide-based additives to improve preventive efficacy beyond simple mechanical sealing. These modifications are clinically attractive because they may reduce bacterial colonization, inhibit biofilm maturation, and promote remineralization of adjacent enamel surfaces.

Rather than a set of isolated observations, the studies on modified sealants converge on a single principle: the biological acceptability of a bioactive additive is concentration-dependent, and increasing antibacterial or remineralizing capacity does not automatically preserve—and can compromise—cytocompatibility [24,25,26,28]. Calcium phosphate, bioactive glass, and polylysine systems were generally well tolerated at optimized loadings, whereas higher concentrations of fluoride-releasing or antibacterial components crossed a threshold beyond which cell viability declined. The practical implication is that additive formulation should target a therapeutic window balancing preventive benefit against cellular tolerance, rather than maximal antibacterial output.

The role of fluoride deserves particular attention. Fluoride release is generally considered beneficial because it enhances remineralization and provides antibacterial activity, which is particularly valuable in high-caries-risk pediatric patients. Glass ionomer sealants and fluoride-containing resin sealants are often preferred for this reason. However, excessive fluoride exposure at the cellular level may induce oxidative stress and has been associated with potential genotoxic effects [18]. The findings of Lai et al. [28] support this concern, suggesting that high fluoride concentrations may compromise cellular viability. Therefore, fluoride release should be optimized rather than maximized, ensuring sufficient preventive benefit without introducing additional biological risk.

The variability among commercial products is best interpreted as a consequence of formulation rather than an intrinsic property of sealants as a class. Cytotoxic potential broadly tracked resin-matrix and filler content, decreasing from unfilled resin-based materials through filled resin-based and resin-modified glass ionomer to silicone-based formulations [13,30]. A higher filler fraction likely lowers the proportion of leachable matrix, while the distinct chemistry and reduced monomer release of glass ionomer-based materials may account for their better tolerance. For the clinician, the relevant message is that the biological profile is governed by material selection, not by the decision to seal itself.

Because residual monomer release is inversely related to the degree of conversion, polymerization quality is a modifiable determinant of biocompatibility that lies directly under the clinician’s control. Importantly, curing adequacy need not be traded against chairside efficiency: reducing light-curing time at an equivalent energy density did not increase cytotoxicity [31]. This is particularly useful in pediatric practice, where shorter procedures aid cooperation—provided that sufficient light intensity, correct tip positioning, and full access to the occlusal surface are maintained to prevent under-curing.

The scarcity of clinical genotoxicity data is arguably the most consequential gap identified in this review. The single in vivo study offers cautious reassurance—overall biocompatibility was good, and the only signal was a transient rise in condensed chromatin for one material at 90 days [27]—but one short-term investigation cannot establish long-term safety. The concern is amplified in children, who may accumulate repeated exposures across multiple restorations over many years, precisely the scenario least represented in the current evidence.

The lack of long-term in vivo studies represents the greatest limitation of the current evidence base. In vitro studies provide important mechanistic understanding, but they cannot fully replicate the complexity of the oral environment, where saliva, pH fluctuations, bacterial biofilm, masticatory forces, enzymatic degradation, and individual host factors continuously influence material behavior. Furthermore, many studies assess cytotoxicity using eluates over 24–72 h, while clinical sealants remain in function for several years. Long-term degradation products, chronic low-dose exposure, and cumulative systemic effects remain insufficiently investigated.

The included studies demonstrate several methodological limitations that should be carefully considered when interpreting the findings of this scoping review. The majority of investigations were conducted in vitro and exhibited substantial heterogeneity in experimental design, including variability in cell types, exposure conditions, duration of observation, and outcome assessment methodologies. In addition, sample sizes were frequently limited and not consistently justified, which may compromise the robustness and reproducibility of the reported results.

Another key limitation is the considerable methodological heterogeneity across studies. Differences in cell lines, extraction protocols, exposure times, material concentrations, and assay methods substantially hinder direct comparability and preclude quantitative synthesis. For instance, fibroblasts, keratinocytes, periodontal ligament cells, and pulp cells may exhibit distinct biological responses to identical materials, while assays such as MTT, XTT, WST-1, LDH, and agar overlay evaluate different and not directly interchangeable biological endpoints. This variability in both biological models and analytical approaches further contributes to inconsistencies in reported outcomes.

Moreover, the absence of standardized protocols for cytotoxicity and biocompatibility assessment complicates the interpretation of findings across the literature. Collectively, these methodological constraints limit the strength of inferences that can be drawn regarding the biocompatibility of pit and fissure sealants. Consequently, the findings of this review should be interpreted with appropriate caution. Future research should prioritize harmonization of experimental designs and adherence to established standards, in order to improve comparability, reproducibility, and the overall quality of evidence.

This review itself also has limitations. As a scoping review, the primary aim was to map available evidence rather than perform quantitative synthesis or risk-of-bias assessment. Only PubMed and Scopus were searched, and although the grey literature was considered, some relevant studies may have been missed. Additionally, older studies often lacked detailed compositional data for sealants, making direct comparison with modern materials difficult. Nevertheless, the strength of this review lies in the comprehensive inclusion of both in vitro and in vivo evidence and the focus on a clinically important but insufficiently synthesized topic.

From a clinical perspective, the current evidence supports the continued use of pit and fissure sealants as safe and effective preventive materials. Their proven role in reducing occlusal caries clearly outweighs the relatively low and mostly transient biological risks reported in the literature. However, clinicians should remain aware that not all materials behave equally. Appropriate material selection, strict adherence to manufacturer instructions, adequate polymerization, surface cleaning after placement, and regular follow-up remain essential steps for minimizing biological risk. Special consideration should be given to pediatric patients, who may be more susceptible to cumulative exposure over time.

Future research should prioritize well-designed long-term clinical studies evaluating cytotoxicity, genotoxicity, endocrine activity, and chronic exposure biomarkers in children and adolescents. Greater attention should also be directed toward BPA-free formulations, bioactive glass systems, calcium phosphate technologies, and other emerging materials that may offer improved safety profiles without compromising retention and caries prevention. Standardized methodologies would greatly improve comparability across studies and strengthen evidence-based recommendations.

Overall, the available evidence suggests that pit and fissure sealants are generally biocompatible and clinically safe, particularly when appropriate materials and proper clinical protocols are used. Nevertheless, residual monomer release, additive concentration, and long-term exposure remain important determinants of biological response, supporting the need for continued development of safer and biologically optimized preventive materials.

## 5. Conclusions

Within the limitations of the available evidence, the included studies generally reported acceptable biocompatibility for pit and fissure sealants used in caries prevention, particularly in pediatric dentistry. Most studies described low to moderate cytotoxicity, primarily associated with resin-based materials containing residual monomers such as TEGDMA and Bis-GMA, whereas glass ionomer-based and silicone-based materials tended to show lower cytotoxic potential. Material composition, degree of conversion, fluoride concentration, and the incorporation of bioactive or antibacterial additives were reported as factors influencing the biological response. Newer bioactive formulations containing nano-calcium fluoride, calcium phosphate, bioactive glass, or antibacterial monomers were associated with favorable biocompatibility in individual studies, although higher concentrations of certain additives were reported to reduce cell viability.

The current evidence base is limited and heterogeneous, consisting predominantly of in vitro investigations, with only one included in vivo human study directly assessing genotoxic outcomes. Consequently, conclusions regarding long-term genotoxicity and systemic biological effects cannot be drawn from the available data. These gaps indicate a need for further well-designed longitudinal clinical studies, particularly in pediatric populations, where repeated exposure to resin-based materials may occur over extended periods. Future research would benefit from standardized biocompatibility assessment protocols, long-term clinical monitoring, and evaluation of BPA-free sealant formulations. Based on the available evidence, material composition, degree of polymerization, and adherence to established clinical protocols appear to be relevant factors in the biological performance of these materials.

## Figures and Tables

**Table 1:** Search Strings.

MEDLINE/PubMed	Scopus
((“Pit and Fissure Sealants”[Mesh] OR fissure sealant*[tiab] OR pit and fissure sealant*[tiab] OR dental sealant*[tiab]) AND (“Biocompatibility Testing”[Mesh] OR “Cytotoxicity Tests”[Mesh] OR biocompatibility[tiab] OR cytotoxicity[tiab] OR genotoxicity[tiab] OR “cell viability”[tiab] OR inflammatory response[tiab] OR “in vitro”[tiab] OR “in vivo”[tiab]))	(TITLE-ABS-KEY(“Pit and Fissure Sealants” OR fissure sealant* OR pit and fissure sealant* OR dental sealant*)) AND (TITLE-ABS-KEY(biocompatibility OR cytotoxicity OR genotoxicity OR “cell viability” OR “inflammatory response” OR “tissue response” OR “in vitro” OR “in vivo”))

**Table 2:** Primary in vivo and in vitro studies.

Authors and Year	Objectives	Type of Study	Material	Cell Line	Type of Assay	Main Findings
**Lertwisitphon et al. (2025) [24]**	To develop ion-releasing and antibacterial resin-based dental sealants and evaluate their biological properties	In vitro experimental study	Experimental sealants based on UDMA/TEGDMA/4-META resin matrix with CQ/DMAEMA initiator. Fillers: SDG = silanated dental glass, MCPM = monocalcium phosphate monohydrate, BAG = bioactive glass, PLS = polylysine. F1: 82 wt.% SDG + 6 wt.% MCPM + 6 wt.% BAG + 6 wt.% PLS; F2: 85 wt.% SDG + 6 wt.% MCPM + 6 wt.% BAG + 3 wt.% PLS; F3: 88 wt.% SDG + 3 wt.% MCPM + 3 wt.% BAG + 6 wt.% PLS; F4: 91 wt.% SDG + 3 wt.% MCPM + 3 wt.% BAG + 3 wt.% PLS; F5: 100 wt.% SDG (control). Commercial comparator: Clinpro™ XT Sealant.	L-929 mouse fibrosarcoma cells	MTT assay	F1 had the highest cell viability compared to other materials (*p* < 0.05). The cell viability of the commercial comparator was similar to that of F2, F3, F4, and F5.
**Fei et al. (2024) [25]**	To develop a novel bioactive pit and fissure sealant containing nano-calcium fluoride (nCaF_2_) and antibacterial monomer (DMAHDM), and to evaluate its cytotoxicity	In vitro experimental study	Novel resin-based pit and fissure sealant modified with nano-CaF_2_ and antibacterial monomer (DMAHDM); compared with Helioseal F^®^ (Ivoclar Vivadent, Schaan, Principality of Liechtenstein).	Human periodontal ligament cells (hPDLCs)	MTT assay	nCaF_2_ did not exhibit toxic effects on hPDLCs at the tested concentration. TEGDMA showed low cytotoxicity, and the cell survival rate with DMAHDM at the same concentration was higher than with Bis-GMA. Monomer toxicity ranked Bis-GMA > DMAHDM > TEGDMA.
**Garcia et al. (2021) [26]**	To formulate Bis-GMA/TEGDMA resin blends and evaluate the biological properties of the experimental resins	In vitro experimental study	Bis-GMA/TEGDMA resin (60:40 wt.%) modified with 2.5 wt.% or 5 wt.% 1,3,5-triacryloylhexahydro-1,3,5-triazine (TAT).	Human keratinocytes (HaCaT)	SRB assay	Incorporation of 5 wt.% TAT into the Bis-GMA/TEGDMA resin induced the lowest cell viability, with no difference between the control group and 2.5 wt.%.
**Gavić et al. (2021) [27]**	To evaluate the cytotoxic and genotoxic effects of fissure sealants on buccal epithelial cells over time (T0–T3, up to 90 days)	In vivo clinical study	Helioseal F^®^, Equia Fil^®^, Constic^®^.	Human buccal epithelial cells (exfoliated cells collected by brushing)	Micronucleus assay (micronuclei, karyolysis, karyorrhexis, condensed chromatin, nuclear buds)	Sealants showed overall good biocompatibility; only Helioseal F^®^ caused a statistically significant increase in condensed chromatin at 90 days, while the other materials showed no significant genotoxic effects.
**Lai et al. (2021) [28]**	To develop a fissure sealant containing chitosan/fluoride microparticles (C/F) with antibacterial activity and fluoride release/recharge ability, and to investigate its biocompatibility	In vitro experimental study	C/F experimental resin (2%/4%) and Clinpro™ fissure sealant.	3T3 fibroblasts	WST-1 assay	Cell necrosis occurred when 3T3 fibroblasts were incubated in the pure extraction solution of 4% C/F and Clinpro™ fissure sealant. The cytotoxicity of 4% C/F was attributed to the negative influence of high fluoride concentration on the cells.
**Koulaouzidou et al. (2018) [13]**	To identify the effect of sealants on the survival of cultured fibroblasts	In vitro experimental study	BeautiSealant (SHOFU, Kyoto, Japan), Clinpro (3M/ESPE, Seefeld, Germany), Conseal F (SDI, Bayswater, VIC, Australia), Grandio Seal (VOCO) and Helioseal Clear (Ivoclar Vivadent, Schaan, Principality of Liechtenstein).	NIH/3T3 mouse embryo fibroblasts	Trypan blue exclusion assay	Cell viability at 24 h and 72 h was lower than in negative controls, and the effect was time-dependent (*p* < 0.05). BeautiSealant, Clinpro and Grandio Seal showed the highest reduction in cell viability in both periods. No significant difference was found between Helioseal Clear and Conseal F (*p* > 0.05).
**Thunyakitpisal et al. (2016) [29]**	To evaluate light-activated pit and fissure resin-based sealant prototypes (LAS-clear and LAS-opaque) for cytotoxicity, compared with commercial light-activated sealants	In vitro experimental study	LAS-clear, LAS-opaque, Delton^®^ clear, Helioseal^®^ clear, Helioseal^®^ opaque, Clinpro™ Sealant.	Human gingival fibroblasts	MTT assay	CL conditioned medium significantly reduced cell viability at 24 and 48 h compared with the control and other sealants (*p* < 0.05). LAS-clear, LAS-opaque, DC, HC and HO conditioned media slightly decreased cell viability.
**Zingler et al. (2015) [30]**	Initial screening of the cytotoxic potential of widely used smooth enamel surface sealants applied to human enamel slices	In vitro experimental study	Composite-based sealants (filled), composite-based sealants (unfilled), silicone-based sealants, and composite-modified glass ionomer-based sealants.	Murine L929 fibroblasts	Agar overlay assay	Cytotoxic potential decreased as follows: unfilled resin-based sealants > filled resin-based sealants > resin-modified glass ionomer-based sealant > silicone-based sealants.
**Wegehaupt et al. (2014) [31]**	To evaluate whether shortening light-curing time while maintaining energy density affects the cytotoxicity of resin-based surface sealants	In vitro experimental study	Seal&Protect (DENTSPLY DeTrey, Konstanz, Germany) and K-0184 (DENTSPLY DeTrey), applied on bovine dentine discs.	Human dental pulp cells and human gingival fibroblasts (HGF)	CytoTox96^®^ Non-Radioactive Cytotoxicity Assay (LDH assay)	Shortening light-curing time while maintaining a similar energy density did not increase the cytotoxicity of the tested sealants.
**Furche et al. (2013) [14]**	To investigate the leaching of ingredients from commercial dental fissure sealers and their cytotoxic effects on human gingival fibroblasts	In vitro experimental study	Helioseal^®^ F, Helioseal^®^ Refill, Fissurit^®^ F, Grandio^®^ Seal, Ultraseal XT^®^ plus and Delton^®^ FS.	Human gingival fibroblasts (HGF)	XTT assay	In eluates from polymerized sealers, comonomers (TEGDMA) and additives were found (e.g., camphorquinone, butylated hydroxytoluene, triphenylstibane). Seven days after the start, the highest amount of TEGDMA was found in the aqueous eluate from Grandio^®^ Seal. The most cytotoxic eluate in the XTT assay was from Fissurit^®^ F.

**Table 3:** Summary of the main biocompatibility findings of the included studies.

Study	Material(s)	Key Biocompatibility Finding
**Lertwisitphon et al. (2025) [24]**	Experimental resin sealants (MCPM + bioactive glass + polylysine)	All formulations biocompatible; F1 (highest additive load) showed the highest cell viability, comparable to or above the commercial comparator.
**Fei et al. (2024) [25]**	Experimental resin with nano-CaF_2_ + DMAHDM	nano-CaF_2_ not toxic to periodontal ligament cells; monomer toxicity ranked Bis-GMA > DMAHDM > TEGDMA.
**Garcia et al. (2021) [26]**	Bis-GMA/TEGDMA resin + TAT (2.5 or 5 wt.%)	2.5 wt.% TAT comparable to control; 5 wt.% significantly reduced keratinocyte viability (concentration-dependent).
**Gavić et al. (2021) [27] (in vivo)**	Helioseal F, Equia Fil, Constic	Good biocompatibility over 90 days; only Helioseal F raised condensed chromatin; no significant genotoxicity otherwise.
**Lai et al. (2021) [28]**	Experimental chitosan/fluoride resin (2%/4%)	4% chitosan/fluoride caused fibroblast necrosis, attributed to excessive fluoride; lower load tolerated.
**Koulaouzidou et al. (2018) [13]**	Five commercial sealants	Time-dependent fall in fibroblast viability; BeautiSealant, Clinpro and Grandio Seal most cytotoxic; Helioseal Clear ≈ Conseal F least.
**Thunyakitpisal et al. (2016) [29]**	Experimental LAS + commercial light-cured sealants	LAS-clear most reduced viability at 24–48 h vs. control; cytotoxic effects overall mild and transient.
**Zingler et al. (2015) [30]**	Resin (filled/unfilled), RMGI- and silicone-based sealants	Cytotoxicity ranked unfilled resin > filled resin > resin-modified glass ionomer > silicone (lowest).
**Wegehaupt et al. (2014) [31]**	Seal&Protect, K-0184 (resin-based)	Shortening light-curing time at equal energy density did not increase cytotoxicity.
**Furche et al. (2013) [14]**	Six commercial methacrylate sealants	TEGDMA and additives eluted (highest TEGDMA from Grandio Seal); Fissurit F the most cytotoxic eluate.

## Data Availability

The original contributions presented in this study are included in the article. Further inquiries can be directed to the corresponding authors.
